# Drinking Water

**Published:** 1896-09

**Authors:** 


### EDITORIAL.

The office of the Business Manager of THE JOURNAL is Nos. 311 and 312 Fitten Building.

The editors are not responsible for views expressed by contributors.

Articles for publication should reach this office not later than the 15th inst.

Address all communications, and make all remittances payable to THE ATLANTA MEDICAL AND SURGICAL JOURNAL, Atlanta, Ga.

Reprints of articles will be furnished, when desired, at a nominal cost. Requests for the same should always be made *on the manuscript.*

### DRINKING WATER.

The necessity for health of a pure supply of drinking water was long ago recognized. For example, the Romans preferred the cost of nine aqueducts connecting the city with the surrounding hills, and furnishing three hundred gallons per head daily, to drinking from the Tiber which flowed through their midst; and there is reason to believe that at a much earlier date the Chinese and Egyptians took the trouble to bore artesian wells of great depth in search of an efficient supply.

The purity of water depends, of course, not only upon its source, but upon its freedom from subsequent contamination as well. One of the first and most important duties of the public officers of cities, towns, or villages, is the careful oversight of the water supply, and in these days immense sums are constantly being spent in connection with this matter. For example, the city of London is now considering the question of abandoning the Thames as a source of supply, and of bringing water for its enormous population from hills many miles way. In like manner all over the world care is being taken to procure a source as pure as possible, and by means of a sometimes complicated series of filters to still further reduce the risks of impurity before it reaches the pipes for distribution. But after it has entered these it is by no means certain that it will reach the consumer uncontaminated. In some places it is doled out on what is termed the *intermittent* system; that is, each house is furnished with one or more cisterns, which are filled during the short fraction of the day in which the water is allowed to run. While this is economical in several respects from the point of view of the water company, it is accompanied by so many dangers to health that as a system it is on the decline. Thus, the water must be stored on the premises, where it is apt to absorb deleterious gases, as in water-closets, etc.; is apt to be stored in improper receptacles, and to be polluted by insects, mice, etc., besides which it becomes comparatively flat and insipid. Cisterns are safe only when frequently cleansed, when made of stone, slate, or galvanized iron, when exposed to the light and air, and covered, and when entirely disconnected directly with water-closets or drains.

While the *constant* system requires continual care of pipes and taps to prevent great waste, and may therefore be expensive, it has the great advantage that no house storage is required except in the water-closet cisterns and a small cistern for the kitchen boiler. Dangers in connection with the constant system exist where water-closets are flushed by a pipe and tap direct from the house main without the intervention of a cistern, for, when the tap is left unscrewed and the water is turned off at the main, foul liquids or air may be sucked up into the pipes and gain entrance to the water-mains. The suction is the result of leakage from the pipes (a common occurrence), which causes a partial vacuum within them. Several epidemics of typhoid fever have occurred in this manner. There is danger in the same way, when water mains and sewers are laid in one trench, that leakage from the sewers may reach the interior of the water pipes when the company's officials turn off the water for some purpose, frequently a daily occurrence.

But the dangers from these services are minute compared to those connected with the use of such shallow wells as are common in many districts, many of which are apparently unprotected at the mouth, and have walls of a porous nature. Seeing that the presence of excrementitious matter may by no means affect the palatability of water, and that a well drains an area surrounding it equal to from fifteen to one hundred and sixty times the depth of the well (depending upon the nature of the soil), it would be safe to assert that a great many persons are consuming daily such suspended or dissolved matters as would greatly disgust them did they know it. Certain it is that all shallow well water, however clear and sparkling, in the neighborhood of human habitations, is suspicious, as are many in more sparsely peopled districts, and that especially in the presence of epidemic diarrhea and enteric fever, before being drunk it should always be subjected to some process of artificial sterilization. In fact, there can be no doubt that the annual mortality figures would be considerably lessened were all waters used only in the cooked condition.

